# Deformable airfoil using hybrid of mixed integration electrolysis and fluids chemical reaction (HEFR) artificial muscle technique

**DOI:** 10.1038/s41598-021-85067-y

**Published:** 2021-03-09

**Authors:** Ramin Zakeri, Reza Zakeri

**Affiliations:** 1 Mechanical Engineering Department, Shahrood University of Technology, Shahrood, Iran; 2 Semnan Science and Technology Park, Shahrood, Iran

**Keywords:** Engineering, Aerospace engineering, Chemistry

## Abstract

In this research, by inspiration of natural myosin motion in artificial muscle contraction, a new method for changing the thickness of an airfoil has been proposed by hybrid of mixed integration electrolysis module and chemical reaction (HEFR) of sodium bicarbonate (NaHCO3 (s)) and acetic acid (CH3COOH (l)). The mentioned method has the ability to create pressure in the fluid in a short time and fast transfer without delay due to the integration of the method in the fluid transfer tube to soft sealed skin. With soft sealed skin swelling and movement of solid skeletal structure, the force is transmitted to the desired mechanism. First, for a single of soft skin and solid structure, remarkable displacement over time in the various loading condition (by the inflation tester) has been investigated. It is shown that the proposed mechanism is capable of moving 246 g during 3 s with total mechanism weight of 10 g. In the following, the mechanism is developed into a symmetrical rhombus (set of soft skin-solid structure) with the ability to contract and expand to provide variable airfoil thickness. The proposed mechanism has the ability to move in the horizontal and vertical axis (expansion and contraction) in lower than 5 s by applying the HEFR technique. Such a mechanism is mounted on a symmetrical airfoil and has the ability to change the airfoil thickness with the appropriate response time. The proposed mechanism can be used in various industrial applications such as robotics.

## Introduction

One of the things that can be seen in abundance in nature is the movement of living organisms, from the movement of sea creatures and Four-legged animal to the flying of birds, using muscle contraction. One of the advantages of muscle contraction in nature is the very light weight, high efficiency, high power and fast response time^1–3^. In man-made devices, for providing the displacement, the rotating or sliding motion of combustion or electric motors have been used which is not so flexible in different conditions^4,5^. For example, an aircraft (A/C) flap, which is controlled by a hydraulic motor, is used to change the aircraft wing surface but compared to wings of birds, this essential part of A/C wings lack of flexibility in various off design conditions^6^. Relationship between airfoil deformation and the issue of artificial muscle is not new to nature, but very little work has been done in this area.

Using several electrical actuation systems is a way to change the trailing edge of airfoil. This method can increase the aerodynamic efficiency from aspects of higher lift/drag ratio and better pitching moment control^7^. Dependency to electrical motors such as servo motor have own limitations and lack of flexibility and also electric motor need extra mechanisms to change rotational forces to axial forces considering high electrical power consumption with low efficiency^4,5,8^. Some authors used airfoil deformation (changing the camber of airfoil) to enhance the aerodynamics performance but the mechanism were not determined^9^. Using smart materials is a powerful option to change the surface of airfoils. The shape memory alloy is a proper choice (SMA) for airfoil control system to maintain Max. Lift/Drag ratio^10^. Shape memory alloy (SMA) is a new method in robotic which using the heat source, a proper strain can be gained^11,12^. One of the drawbacks of this method is dependency to heat and cold source and response time is not so short. Also, the response is non-linear and control of strain is so complicated^10,11^. Considering other smart materials such as PVDF polymer or piezoelectric ceramic, they have very low strain rate^10,13^. Apart from a few limited methods used in airfoil deformation, practical methods with significant benefits in artificial muscle have been researching recently. For example, dielectric elastomer provides conspicuous displacement in short time by changing the electric current to mechanical energy^11^. Some scholar applied the artificial muscle based on the dielectric elastomer method for displacement of robot like a cuttlefish robot^14^. Some of these methods including electroactive polymers (EAP), HASEL have some disadvantages such as requiring high voltage, far from natural mechanism and low total efficiency^15,16^. Using some smart materials which is influenced from lighting and show a small displacement, has a weak output force (mN) and response time is not short^17,18^.

The conversion of chemical energy into mechanical energy may be one of the most successful methods of generating sufficient power to perform a functional displacement, because in nature such a method is widely used in living things. Martinez^19^ used the chemical reaction between glucose and oxygen to release enough electrical energy and transfer it to an electro active polymer for a significant displacement of an artificial muscle. Considering the limitation of EAP, changing chemical energy to electrical energy and then to mechanical energy will result of low efficiency. Some traditional methods, such as hydraulics or pneumatics, have advantages such as sufficient high-efficiency power and have been used in artificial muscle. It is noteworthy that some methods, such as Fluid-driven origami-inspired artificial muscles have the advantage of converting pressure to mechanical work with high efficiency, but the need of high-pressure compressor or electric motor would be one of the main the disadvantages of this method^8,20,21^.

With a closer look at the nature as a complete reference of proper engineering performance and advantages, one of the mechanisms that may exist in myosin movement is thin membrane swelling such as a thin bag swelling and its momentum will transfer to the myosin stem and consequently myosin stem motion will move an actin period. This probable rule has been inspired for the present paper.

In such a way that the use of pressure conversion mechanism to mechanical action has been used in soft sealed skin or sealed bag (similar to hydraulic system) and movement of soft skin is transferred to solid skeletal structure. The source of pressure in natural myosin comes from electrical stimulation by synapses and chemical reactions (similar to hybrid of electrolysis and chemical reaction method)^1,22,23^. In this article, inspired by nature, a combination of electrical stimulation and electrolysis methods, as well as chemical composition, etc. are used to produce pressure, and the action of soft skin swelling causes expansion or contraction. Pressure conversion to mechanical work is used to deform the airfoil (change the thickness). In this article, concentration is more on how to provide a remarkable displacement and the study of aerodynamic efficiency has not been considered.

## Results and discussion

In this section, the results, obtained from the combination of the HEFR technique and deformable airfoil, are presented; first, by considering the new proposal method for electrolysis using mixed integration module, for a single soft skin-solid structure member, the angular rotation over time in different form including pure electrolysis, chemical reaction and HEFR are investigated, and then the results will be developed for a complex of multi- soft skin-solid structure member with ability of both contraction and expansion. Finally, the results from changing the size of the diamond body have been used to change the shape of the symmetric airfoil.

### Relation between natural muscle contraction and applied HEFR technique for active deformable airfoil

**Figure 1:**
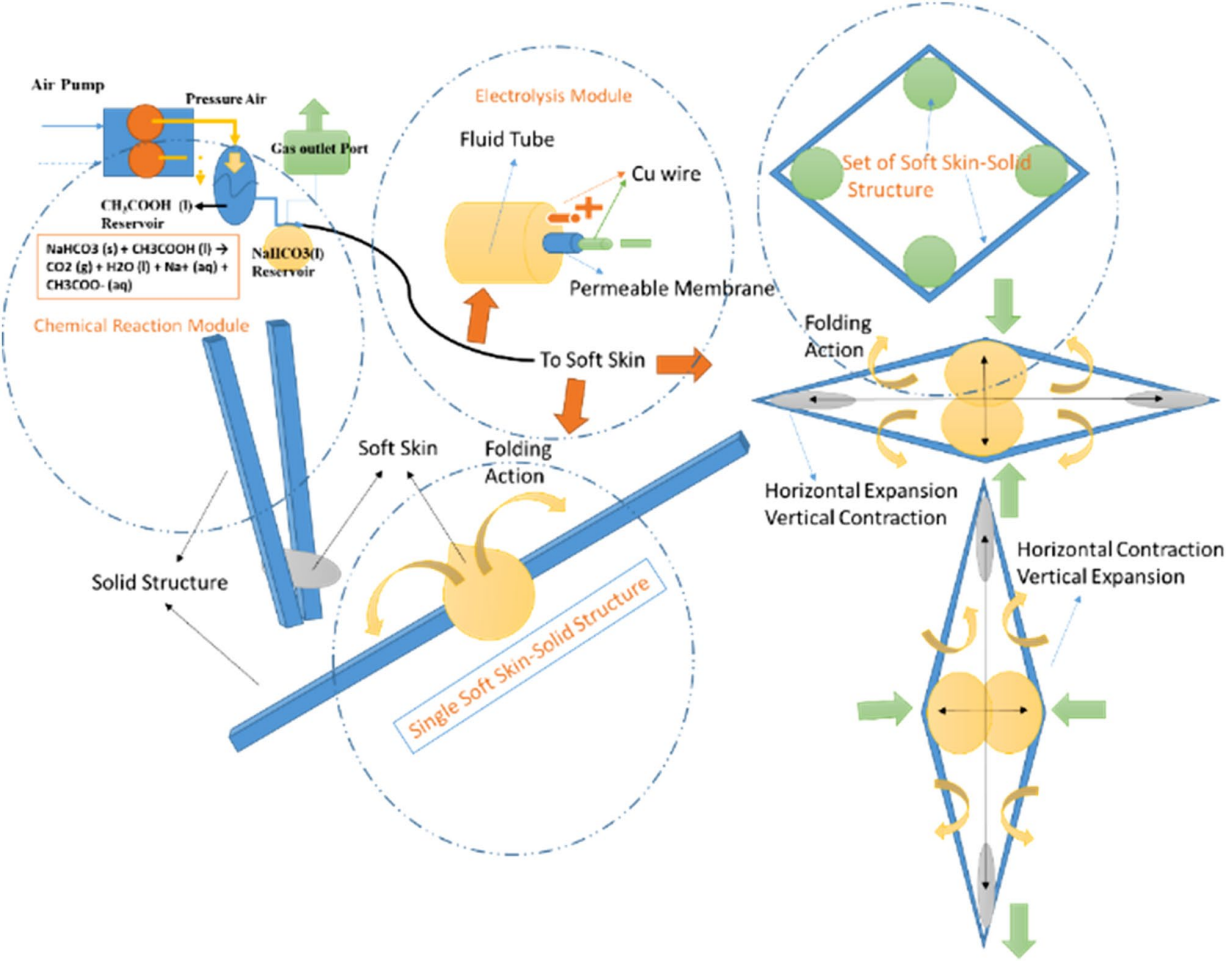
Principle of operation of HEFR in single soft skin-solid structure mode (just folding action in one direction) and set of soft skin-solid structure (bidirectional folding action).

The main idea of this study, inspired by myosin-actin in a fiber of muscle contraction in nature and the similar artificial mechanism, is applied to a flexible airfoil for gaining the volume changes of airfoil. The contraction of a muscle in nature, based on sliding filament theory, begins with the stimulation and movement of myosin by an electrical signal from motor neuron—central nervous system (CNS), called action potential and consequently by releasing calcium from the sacomplas and changing of ATP (Adenosine tri-phosphate) to ADP (Adenosine diphosphate) the energy is released and the myosin will move like a wavy motion. The result of the wavy motion will be the connection to actin (bridge cross) and the pulling of actin, and a contraction will occur. By reducing the released energy, myosin will return to its original state and the muscle will relax. As be shown in Fig. 1, the proposal mechanism uses the mixed integration electrolysis module and power of chemical reactions to be obtained fast and strong displacement^1–3^.

The proposed mixed integration electrolysis module is a state of electrolysis in which two electrodes are separated by a thin permeable membrane and the ions are released as a mixture and the fluid pressure rises in a short time. This electrochemical process is similar to stimulation of myosin in the natural muscle by electrical signal from CNR and chemical reaction of calcium and the release of energy. The pressurized fluid moves and causes the soft skin or artificial myosin to swell, and the filled bag causes the angular rotation of solid structure or artificial actin. The combination of soft skin and solid structure, as be illustrated in Fig. 1, can form a variable diamond-shaped object that is similar to symmetrical airfoil and has important advantages such as ability to expand or contraction motion. Therefore, by establishing the electric current of mixed integration electrolysis process, the fluid pressure increases and the pressure in the soft skin causes swelling and solid structure movement. In a rhombus-shaped object, the two soft skins facing each other are stimulated together to expand and two other facing soft skins should be relaxed, thus rhombus will be expanded in horizontal direction and get contraction in vertical direction and this process can easily be reversed by changing the expansion of two other soft skins and relaxing those soft bags that had expanded, consequently rhombus (symmetric airfoil) will displace in vertical direction and gain contraction in horizontal direction.

## Materials and methods

**Figure 2:**
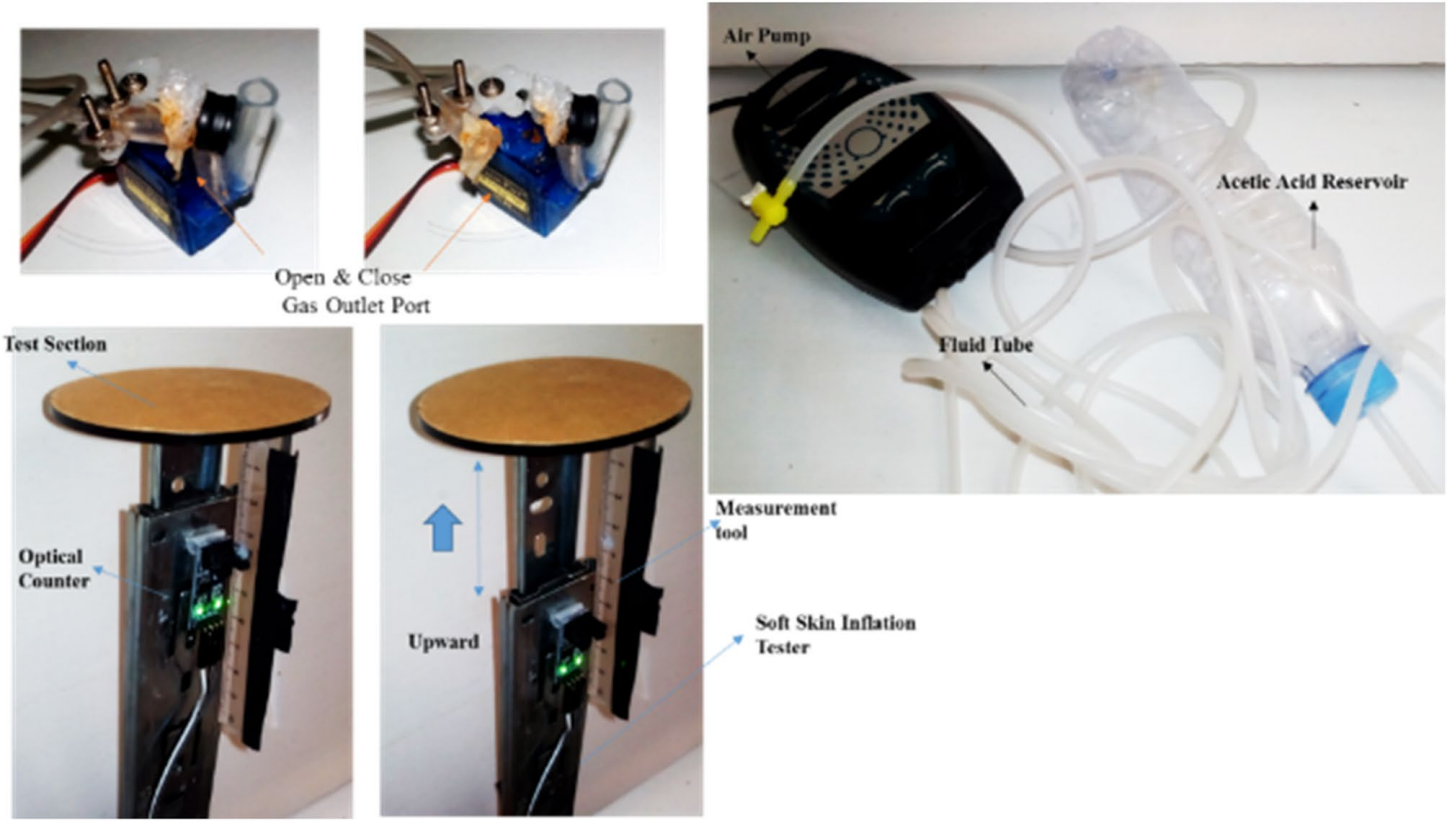
Test equipment including Gas outlet port for depressurizing the swelling soft skin, pump for flowing fluid (acetic acid) through fluid tube and inflation tester for measurement of displacement over time.

The manufacturing process including used materials, fabrication and tests can be categorized into three sections as follows:

1. Electrolysis module section: The manufacturing technique in this section is proposed for the first time in this article, which is called the mixed integration electrolysis module. This section consists of two electrodes made of copper or tin, so that the diameter of the electrode is 0.4 mm and the diameter of the rubber tube in which the fluid is flowing in it, is 5 mm with a wall thickness of 1 mm. One of the electrodes is placed in a very thin layer of cotton mesh with a thickness of 0.1 mm and is wrapped around the wire by a rotating device so that one of the electrodes has a permeable coating. Now both electrodes should be placed in the fluid tube line and two poles should be extracted from fluid tube line wall. The fluid flows through the pump and as soon as it enters the electrolysis line, due to the proximity and relative elongation of the electrodes, a suitable electrolysis action occurs with regard to the mentioned electrolytic composition. one of the limitation of this method is that electrolysis process should be carried out in modular form and in small scales similar to real cells, there are all three combinations of hydrogen, oxygen and heat in the tube line, like the action of burning food in the cell, but due to the low amount of gas produced and the low heat of combustion, it never takes place and we can use the high advantage of proper production power.
2. Soft sealed skin-solid skeletal structure actuator section: This section consists of a small airbag that is located between a hinged solid structures. Soft skin is attached to the rubber air tube line and consists of three different layers. The first layer is a very thin layer in the form of tears and is completely impermeable to the outside, but it is very sensitive to rupture. This layer is made of very thin plastic. The second protective layer is made of transparent adhesive tape, which will prevent damage and rupture of the previous layer due to increased pressure. The third layer of transparent adhesive tape attached to solid structure and soft skin is thicker and provides a protective action and binding to hinged strips. Solid structure is a hinge from two aluminum bars with width of 10 mm and 40 mm in length. Note that when connecting the impermeable soft skin to the fluid line, non-leakage adhesive must be used and it is very important to ensure no leakage, because in practice the pressure in these points is high and in case of the slightest leakage of soft bag and solid structure does not work, especially if the system is under load.
3. Test equipment and accessories: To perform mentioned experimental testes, there is a need for a pump from reservoir to flow a fluid to electrolysis tube line. The pump used is of the piston type with a maximum output flow of 5 ml per second. Working fluid based on the several experiences is a combination of sodium bicarbonate (NaHCO3 (s)) and acetic acid (CH3COOH (l)). The released pressure system, which includes a small out flow valve and a servomotor model (SG90) to close or open the valve. Optical method has been used to measure displacement in this study. The applied sensor is ‘OPTO COUNTER MODULE 5MM’ which, by moving a transparent ruler attached to sliding section, dark points are recognized from transparent and by counting of difference between dark and transparent, amount of displacement with accuracy of millimeter are calculated which is Shown in the Fig. 2.

### Deformation of soft sealed skin-solid skeletal structure

Considering the mechanism described in the previous section, it is necessary to increase the pressure in the fluid to inflate the soft sealed skin. In this section, we first examine an element of soft skin and solid structure and examine it in two modes: pure electrolysis and the hybrid of electrolysis and chemical reaction. In the next step, a complete expansion contraction mechanism is presented which is responsible for changing the thickness of an airfoil.

### Deformation of single soft skin-solid structure by pure electrolysis

**Figure 3:**
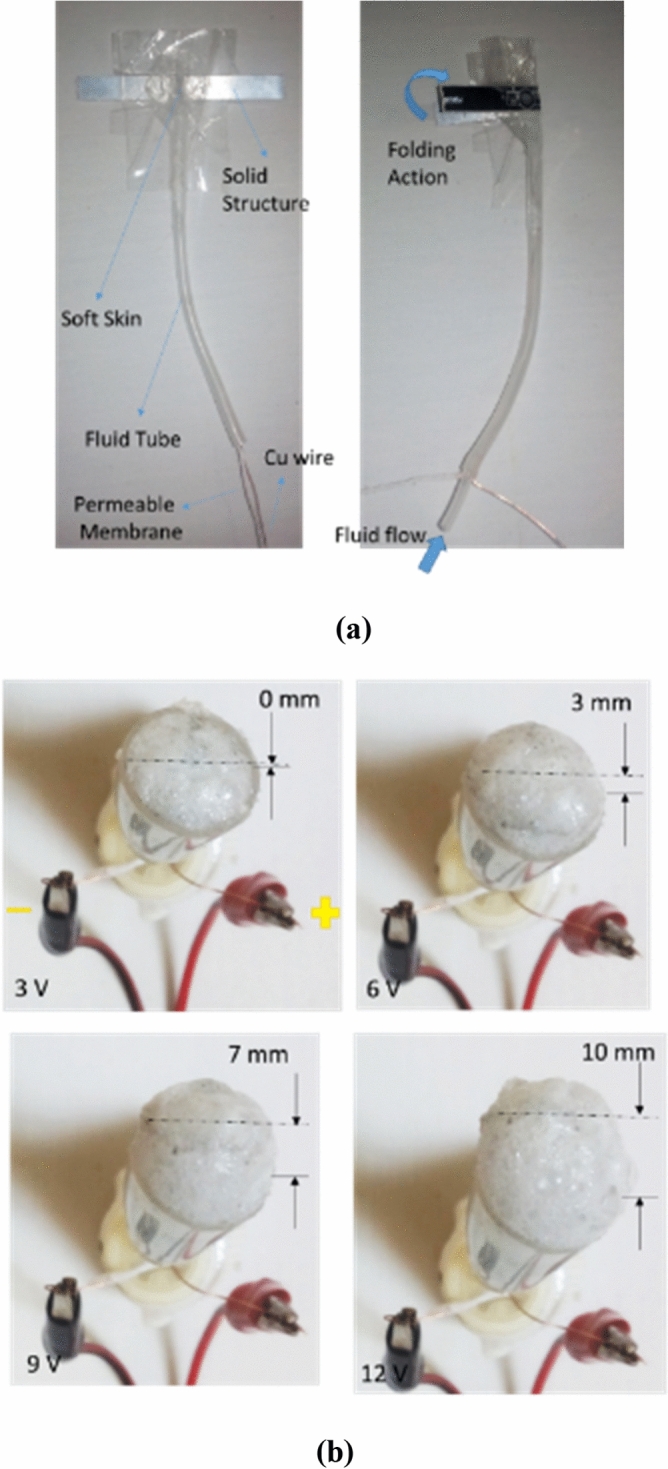
Mixed integration electrolysis module. Fabrication of single soft skin-solid structure with mixed integration electrolysis module **(a)**, chemical reaction of electrolysis through electrolyte in different voltages **(b)**.

In this section, we will examine the effect of electrolysis and soft skin swelling and finally solid structure movement. In this paper, mixed integration electrolysis method is used for the first time. This method involves placing two strands of copper wire with a thin membrane between them in the fluid carrier tube to the soft bag. With the passage of electric current due to the proximity of the two electrodes and the length of the electrodes, a proper electrolysis is created and the fluid pressure will rise rapidly. The desired solution for electrolysis is a combination of NaHCO3 (s) and CH3COOH (l) (mixed them and let to be relaxed which is called inactive mode) which is entered into the fluid tube by the pump and lines are equipped with mixed-integration electrolysis module on a small scale, the pressure enhance promptly and causes soft skin swelling and solid structure movement. Also, the geometry of a soft skin-solid structure element and the mixed-integration electrolysis model are given in the Table 1. In Fig. 3a,b, the detail of mixed integration electrolysis module and the performance of electrolysis in different voltage is shown. As can be seen, by increasing the voltage, volume of discharged gases raise because of more decomposition of fluid molecules. This increase in volume or constant volume increase in pressure will cause soft skin to swell.

**Table 1:** Characteristics of single soft skin-solid structure member.

Length of electrolysis module	120 mm
Materials of electrode	Cu
Length of solid structure from pivot	40 mm
Diameter of soft skin	15 mm
Material of membrane	Cotton
Rate of fluid flow	1 mL/s
Ration of CH_3_COOH to NaHCO_3_	1/10

**Figure 4:**
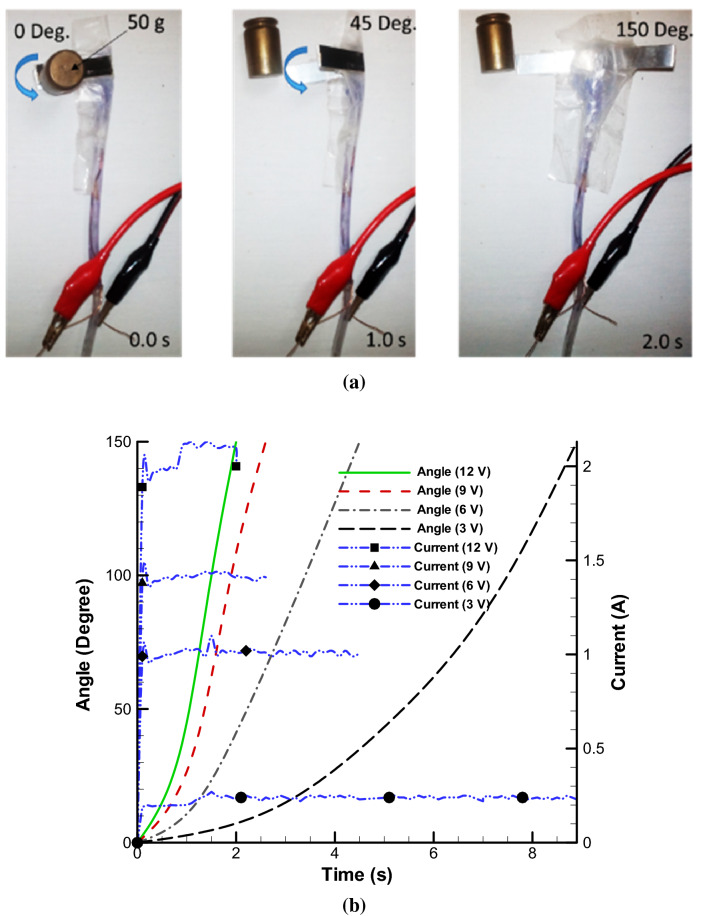
Angular rotation of single soft skin-solid structure without any loading condition. Angular rotation of pure electrolysis of single soft skin for 2 s **(a)** in 12 v. Plot of various angular rotation over time in different voltages **(b)**.

Figure 4a shows combination of single soft sealed skin and folded solid stripes which, by applying integrated electrolysis mod in 12 V, it can rotates over period of almost 2 s and have enough power for easily throwing away 50 gr weight. By swelling the artificial myosin or soft bag, artificial actin or solid skeletal structure tend to rotate very fast and transfer the momentum. In the Fig. 4b, the angular deformation plot of a soft-solid element with respect to time is given in different voltages without any loading condition. Undoubtedly, increasing the voltage is an important factor in faster decomposition and breaking of molecular bonds and obtaining faster response time and stronger displacement. Also, amount of current consumption increases nonlinearly. For example, by selecting 12 v, response time become faster more than 4.5 times compared to 3 v and current consumption enhances more than 8 times. The choice of 12 V is because raising the voltage further can damage the mechanism and speed up the corrosion of the electrodes and also the total efficiency will decline sharply. This paper does not examine the efficiency, corrosion of electrodes and their lifespan.

#### Deformation of single soft skin-solid structure by hybrid electrolysis and active fluid chemical reaction (HEFR)

Sometimes it is necessary to perform the fast and strong expansion and contraction operation, such as the jumping of a living creature or the rapid reaction of changing the surface of the airfoil. In these cases, the combination of chemical reaction and electrolysis will have such an advantageous feature. In other words, the action of the active chemical compound (active mode) in the fluid line has been injected and then the electrolysis is added continuously to increase volume of discharge gases. The volume of gas produced is much higher than the volume of soft bag, and significant power and speed are achieved in a fraction of the time. One of the effective parameters in the amount of displacement during the proper response time is the resistance force against motion. This resistance force includes all the factors that resist the contraction or expansion through motion and tend to prevent from any changes. To test the capability of the proposed method, a tester mechanism has been developed which records the amount of displacement by sliding upwards. The method of recording numbers is optical, which by recording the displacement status by counting the difference between dark points and transparent, the displacement amount is recorded with an accuracy of millimeters.

**Figure 5:**
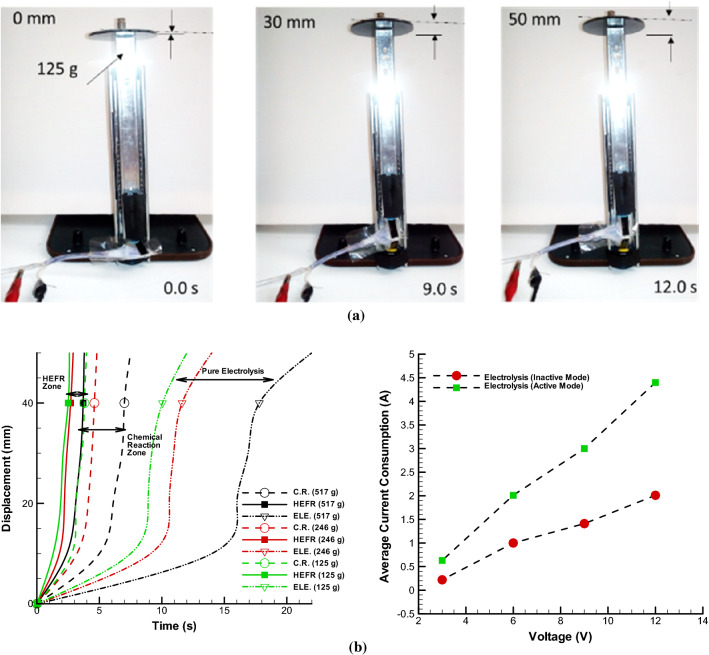
Sliding displacement of single soft skin-solid structure over time. Movement of sliding displacement of inflation tester over time in pure electrolysis mode in 12 v **(a)**. Plot of displacement over time in modes of pure electrolysis (12 V), chemical reaction and HEFR in different loading condition and average current consumption by changing the voltage for two different active and inactive modes **(b)**.

In the Fig. 5a, the movement of a soft-solid set under 125 gr load is shown using inflation tester over time. As it is proved, the proposed method has ability to show a conspicuous displacement in proper response time for example, the proposed single soft-solid has 50 mm displacement in 12 s in pure electrolysis mode while total weight of a fiber is 10 gr. Further investigation in the Fig. 5b illustrates the trend of displacement over passing time, considering various conditions including pure electrolysis, chemical reaction and HEFR, under different loading condition. As can be seen, There are three distinct regions where the range of pure electrolysis has the longest time to change position, the region of pure chemical reaction has the lower time for soft skin swelling (about 67% percent lower than the pure electrolysis) and the HEFR region has the lowest displacement time (about 80% lower than to pure electrolysis). Also, with increasing load, the displacement time increases nonlinearly. In other words, if the load is too much, the number of modules should be increased and the load should be divided. In this study, the weight of 246 gr for such a mentioned module (Table 1) is appropriate and takes about 2.9 s for HEFR mode, which is suitable for the application of changing the position of a small airfoil. Each state change consists of three stages. In stage one, it will take a relatively long time for slow and linear changes to occur. In the rapid reaction stage, an instantaneous state change occurs and in the final stage, the changes decrease slowly. It is noteworthy that the time of displacement changes can be increased or decreased according to the effective parameters, for example, by increasing the voltage or the presence of more chemical compounds (more active state) or longer electrolysis module can be achieved much faster time and without considering the limitations the structure the electro-chemical energy conversion will be properly converted to mechanical without restriction. The purpose of active state is that the chemical reaction is instantly carried out and turned into work. As can be seen, the chemical reaction has the ability to produce gases in a short time, and the combination of this state with electrolysis results in a much faster reaction. The investigation of this research shows that almost without electrolysis, proper reaction occurs but consumption of chemical action is so high and most of releasing energy should be out via outlet valve. By applying pure electrolysis, slow contraction–expansion is formed and hybrids of them give very fast and strong reaction, almost two times shorter compared to pure chemical reaction.

### Deformation of multiple soft skins-solid structures and deformable airfoil

In order to develop the present method, for various applications from robotics to aerospace, it is necessary that expansion and also contraction occur in a set of soft skin-solid structure. Such a mechanism in nature would cause the movement of one muscle in one direction as well as the contraction of another muscle in the opposite direction. In this paper, the distribution of four artificial myosins or soft bags at four angles of a rhombus is suggested. Swelling of the two reciprocal artificial myosins compresses the other two reciprocal soft skins and, conversely vice versa. By changing the thickness of rhombus, this method can be attributed to changing the thickness of the symmetric airfoil, which is very useful for producing different and intelligent lift force.

**Figure 6:**
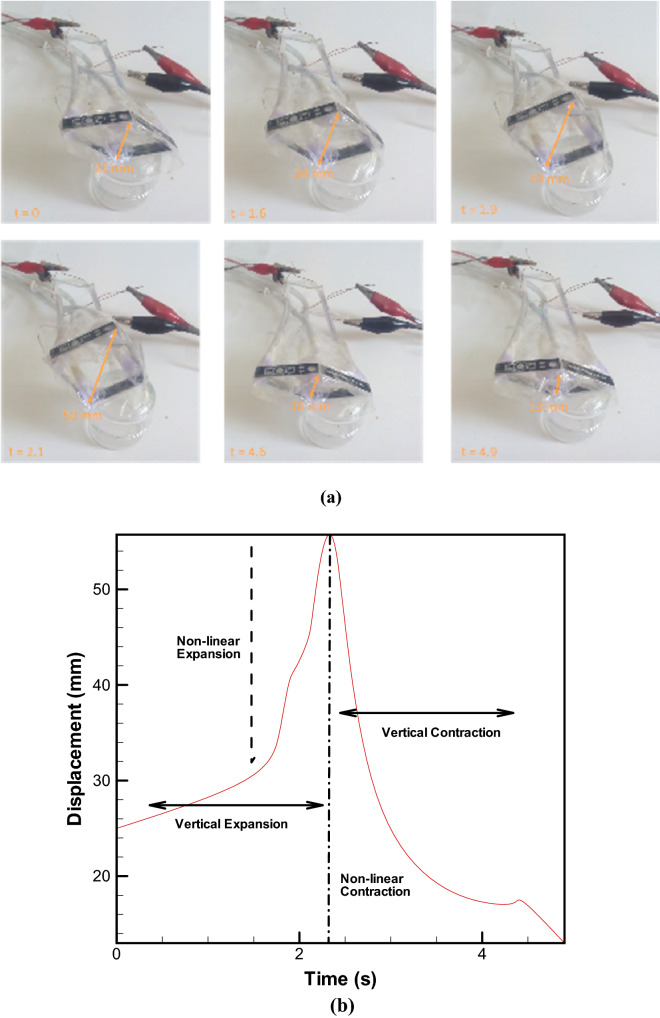
Displacement of set of soft skin-solid structure over time. Vertical and horizontal displacement of a set of soft skin-solid structure using HEFR technique **(a)**. Plot of vertical displacement (thickness of airfoil skeleton) over time **(b)**.

**Figure 7:**
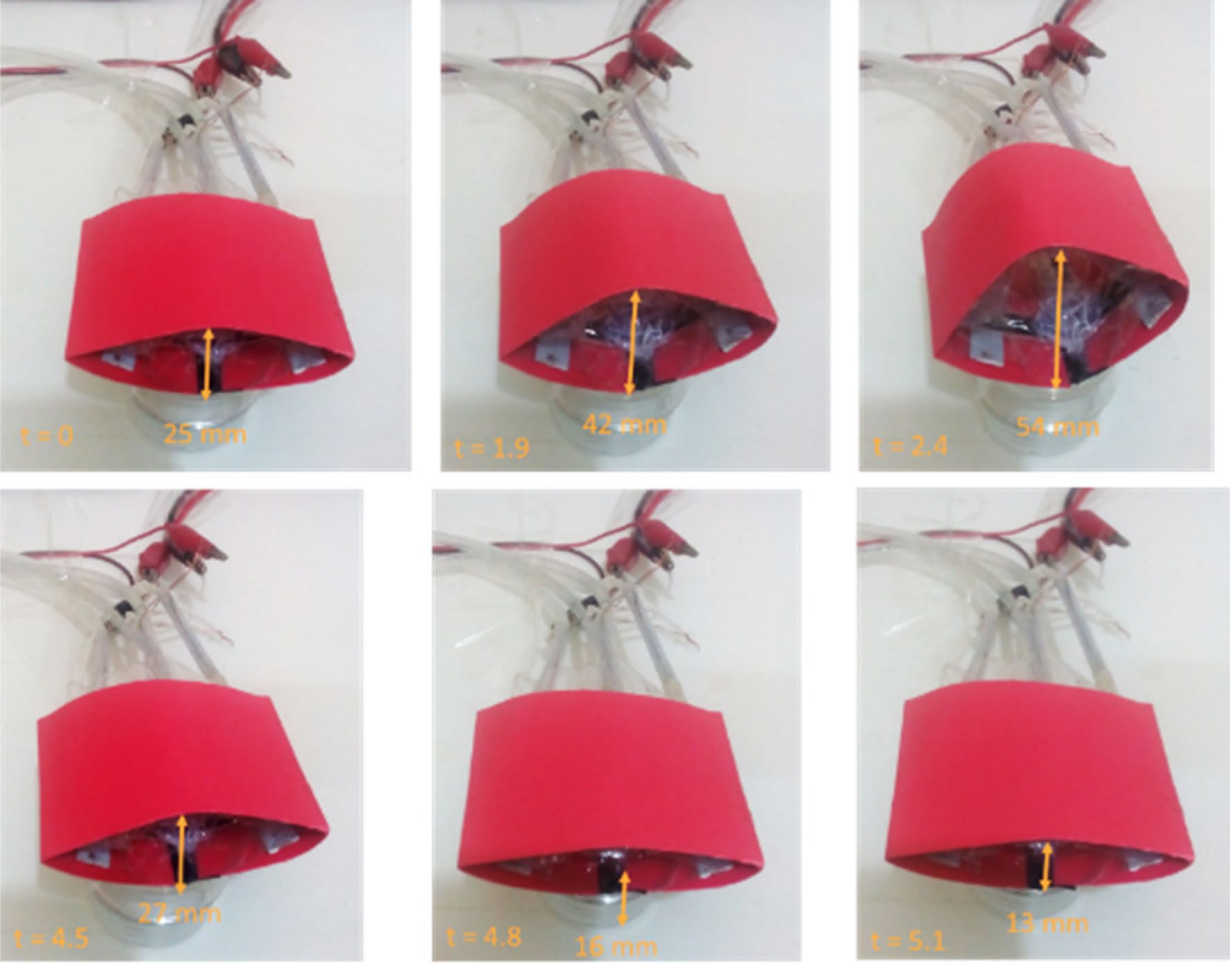
Variation of thickness of small airfoil over time using HEFR technique.

In the Fig. 6a, the structure of airfoil skeleton and performance of this proposal are shown using HEFR technique. Electrolysis has been used in an integrated manner, as mentioned. With the entry of the chemical into the active or inactive state, the electrolysis action causes the mixture to decompose and change to gases. As can be seen, expansion movement in horizontal direction and contraction in vertical direction and vice versa would be possible by proposal mechanism. Vertical displacement of proposal mechanism is enhanced over time (2 s) by two opposed soft skin- solid structure and again is decreased quickly beyond original size by other two soft-solid element. Figure 6b depicts a trend of variation of thickness over passing time. As can be observed the slow linear slope occurs at the first moment (before 2 s) and then a sharp jump is formed due to high volume released gases and then by discharging gases and pressurizing the other soft bags, a fast decreasing in thickness is happened. This method of changing the airfoil skeleton has been applied in changing the surface of an airfoil, which is shown in the Fig. 7. By changing the thickness of the airfoil, all its aerodynamic properties based on the ref. 6 will change. Comparison of mentioned method with other methods are presented in Table 2. Without considering the chemical reaction and gas production, conspicuous inflation will not be achieved but by using the integrated electrolysis module and a chemical reaction (HEFR) with a very low fluid rate of less than 1 mL/s (diameter of a soft bag would be almost 15 mm), remarkable Inflation occurs very quickly, while if we only want to inflate the bags at such a low flow rate without any gas production, it will be so inefficient and also we have not enough power to inflate under loading condition. Proposal method is a proper method for changing the airfoil surface from thickness to camber or other applications such as robotic moving parts.

**Table 2:** Comparison of hybrid Mixed integration electrolyze module and fluid chemical reaction (HEFR) with other methods.

Method	Advantageous	Disadvantageous	Ref
HEFR	Extremely high swelling ratio with short response time due to using mixed integration electrolysis method: SR: 0 to more than 2 RT: 0.5 s to desire value System can be adjusted to desired value without some limitation such as stopping the displacement due to high loading condition Closer performance and mechanism to the real nature Extremely low price and weight, high power to weight ratio and simple for manufacturing	Need equipment and chemical materials for expansion or contraction	–
HASEL	High swelling ratio and short response time: SR: 0 to 1 RT: 0.5 s to desired value No need any special equipment to work	Need high voltage	^14,15,16^
Pneumatic A. M	High swelling ratio and short response time: SR: 0 to 1 RT: 0.5 s to desired value	Need high power and heavy compressor, noise pollution	^8,20,21^
Hydrogels	Used soft materials similar to real muscle, high swelling ratio: SR: 0 to 6	Long response time RT: more than 10 s	^24^
Electroactive polymers	Compact and light structure, high swelling ratio and short response time SR: 0 to 1 RT: 0.5 s to desire value	Need high voltage	^25^
Shape memory alloys	Can be used without electricity Swelling ration of 1	Need equipment and Need heating and cooling system and long response time more than 10 s	^10–12^
Elastomer	High swelling ratio and single self-contained mechanism	Long response time more than 10 s	^11^

## Conclusion

In this paper, a new solution for deforming the thickness of a flexible airfoil by mixed integration electrolysis method was investigated. The present method, based on the study of the physiology of the muscular organ of the creature, developed for a set of soft sealed skin-solid skeletal structure in different states of pure electrolysis, pure chemical reactions and a hybrid of them. It was shown that considering mentioned geometry and scales the present mechanism with a weight of 10 g, under 246 g load, could provide 50 mm displacement in 3 s as an applicable range. Using a combination of soft and solid elements, a variable diamond shape was developed that was deformable in the horizontal and vertical axes. The results of moving different diameters over time and finally changing the thickness of a symmetrical airfoil were presented in which complete expansion and contraction of thickness of airfoil occur in 5 s. The results showed that the use of the combined method will have the advantage of sufficient power compared to the high operating speed and proper control. In the case of pure electrolysis, the reaction rate is lower, but the use of chemicals is very small. In the combined state, we will have high chemical consumption but high strength and reaction. The present method can be extended to other industrial applications such as robotics.

## Data Availability

The authors declare that the data supporting the findings of this study are available within the paper and additional data on methods used are available upon reasonable request.
